# Evaluating predictions of the patterning cascade model of crown morphogenesis in the human lower mixed and permanent dentition

**DOI:** 10.1371/journal.pone.0304455

**Published:** 2024-06-27

**Authors:** Dori E. Kenessey, Christopher M. Stojanowski, Kathleen S. Paul

**Affiliations:** 1 Department of Anthropology, U niversity of Nevada, Reno, Nevada, United States of America; 2 Center for Bioarchaeological Research, School of Human Evolution and Social Change, Arizona State University, Tempe, Arizona, United States of America; 3 Department of Anthropology, University of Arkansas, Fayetteville, Arkansas, United States of America; Universidad Nacional de la Plata Facultad de Ciencias Naturales y Museo, ARGENTINA

## Abstract

**Objective:**

The patterning cascade model of crown morphogenesis has been studied extensively in a variety of organisms to elucidate the evolutionary history surrounding postcanine tooth form. The current research is the first to use a large modern human sample to examine whether the crown configuration of lower deciduous and permanent molars aligns with expectations derived from the model. This study has two main goals: 1) to determine if metameric and antimeric pairs significantly differ in size, accessory trait expression, and relative intercusp spacing, and 2) assess whether the relative distance among early-forming cusps accounts for observed variation in accessory cusp expression.

**Methods:**

Tooth size, intercusp distance, and morphological trait expression data were collected from 3D scans of mandibular dental casts representing participants of the Harvard Solomon Islands Project. Paired tests were utilized to compare tooth size, accessory trait expression, and relative intercusp distance between diphyodont metameres and permanent antimeres. Proportional odds logistic regression was implemented to investigate how the odds of greater accessory cusp expression vary as a function of the distance between early-developing cusps.

**Results/Significance:**

Comparing paired molars, significant differences were identified for tooth size and cusp 5 expression. Several relative intercusp distances emerged as important predictors of cusp 6 expression, however, results for cusp 5 and cusp 7 did not match expected patterns. These findings support previous quantitative genetic results and suggest the development of neighboring crown structures represents a zero-sum partitioning of cellular territory and resources. As such, this study contributes to a better understanding of the foundations of deciduous and permanent molar crown variation in humans.

## Introduction

Dental anthropological analyses often utilize nonmetric crown traits to assess the degree of similarity among global populations [1–17], detect biological kinship within a site [18–29], establish phylogenetic relationships across extinct and extant hominoid taxa [30–49], explore the population history associated with specific groups or geographic regions [50–70], and more recently, to estimate population affinity in recently deceased individuals as part of the biological profile [71–82]. The nonmetric crown traits dental anthropologists rely upon to conduct these analyses include the presence and expression of accessory cusps. Accessory cusps commonly occur in the posterior dentition (*i*.*e*., premolars and molars) and begin to develop after the initiation of primary cusp formation [83–85]. In human molars, the early-developing primary cusps are the a) paracone, protocone, and metacone (upper molars), and b) protoconid, metaconid, hypoconid, and entoconid (lower molars) [84–91]. Late-developing upper molar accessory cusps may include the hypocone, Carabelli’s trait, cusp 5 (*i*.*e*., distal accessory tubercle or metaconule), and less frequently, the parastyle (*i*.*e*., paramolar tubercle), all of which occur on the periphery of the earliest forming cusps. Lower molar secondary cusps are more centrally-located, often positioned between early-forming cusps, and include cusp 5 (*i*.*e*., hypoconulid), cusp 6 (*i*.*e*., *tuberculum sextum*), cusp 7 (*i*.*e*., *tuberculum intermedium*), and less frequently, the peripheral protostylid [87, 89, 92–94].

Crown morphogenesis is the crucial embryological time frame during which the shape, size, and relative position of these accessory cusps are defined. Tooth cusp position first materializes with the appearance of the primary enamel knot (PEK) during the cap stage of dental development at the tip of the developing tooth bud [95–97]. The PEK is a transient structure formed by a cluster of tightly packed epithelial cells as a result of mesenchymal *BMP4* and *EDAR* expression [96, 98–100]. While the PEK does not directly map out cusp shape, size, and patterning, it has a role in inducing secondary enamel knot (SEK) production, and defects in PEK shape and size have important downstream consequences for final tooth form [97, 99, 101–103]. At the end of the cap stage, *MSX2*, *BMP2*, *BMP4*, and the cell cycle inhibitor P21 terminate PEK cell proliferation and initiate its apoptosis [98, 104, 105]. Prior to PEK dissolution, this structure prompts the formation of SEKs, an event that marks the beginning of the bell stage of dental development [97, 99, 103, 106–109].

SEK placement is an indicator of future cusp tip location through aggregate activator signaling, which promotes epithelial cell differentiation into non-proliferative knot cells (*e*.*g*., *FGF4*, *SHH*), and inhibitor signaling, which represses differentiation and instead stimulates mesenchymal growth in the tooth germ (*e*.*g*., *BMP4*, *WNT6*) [106, 110–112]. The complex interaction between activators and inhibitors determines the rate of proliferation across different regions of the epithelium and mesenchyme. Since the stellate reticulum prevents tissue expansion toward the root, mesenchymal proliferation induced by inhibitor signaling causes a lateral expansion in the surrounding epithelial tissue of the crown [87, 106, 113–115]. Spatial differences in cell proliferation rates around SEKs eventually cause inner enamel epithelial folding due to the steady increase in the area of the dental papilla within the spatial constraints of its surrounding enamel organ [87]. This interactive signaling network among the SEKs ensures appropriate cusp arrangement culminating in a functional tooth crown. Importantly, this signaling network governs the spatial organization and cusp pattern exhibited in molars, resulting in species-specific crown morphology [107, 116, 117].

Evolutionary developmental (evo-devo) models have been proposed to explore the evolvability of tooth form through dental development and elucidate the evolutionary history underpinning the taxonomic diversity seen in crown morphology [93, 116, 118, 119]. The patterning cascade model (PCM) specifically focuses on explaining molar crown morphogenesis [116]. The PCM highlights the complex interaction across developmental pathways through signal activation and silencing that work in tandem to shape the crown, resulting in cumulative morphological differences with each iteration. This ultimately manifests as distinct morphological patterns in posterior teeth. The model posits a higher likelihood of late-developing accessory cusp presence and/or a greater degree of accessory cusp expression with: a) shorter distances between earlier-developing cusp tips (*i*.*e*., each successively appearing SEK is located directly outside of the inhibition zone of preceding SEKs), b) longer periods of crown development (*i*.*e*., larger crown), and c) more cells available for successive cusp development [107, 114–116, 120].

**Fig 1:**
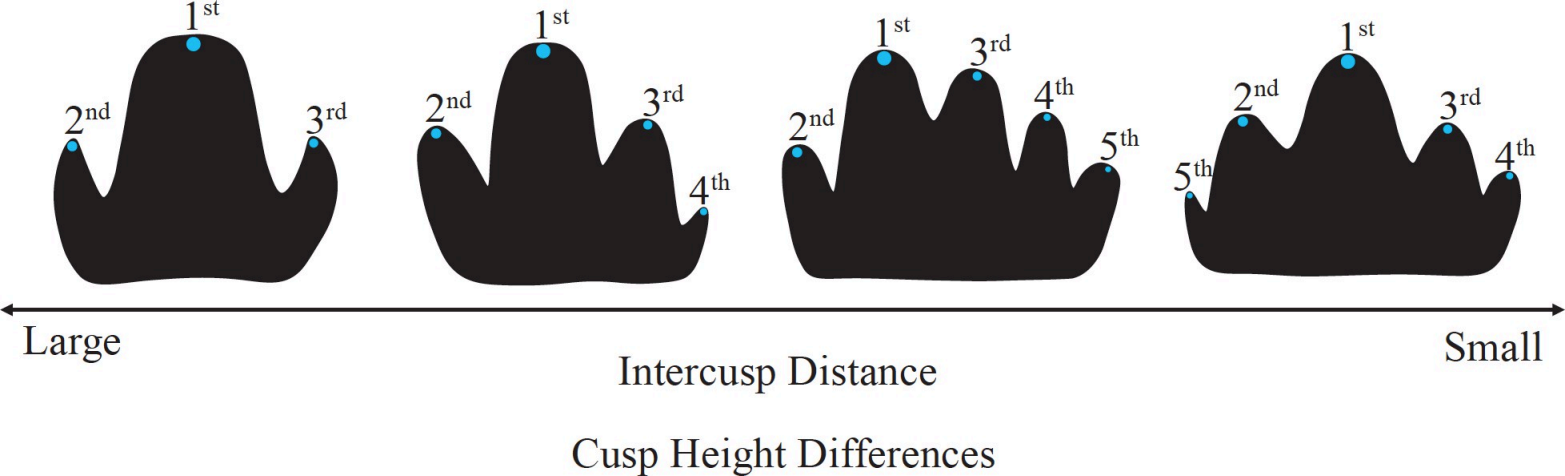
Morphological variation of Lake Ladoga seal molars following predictions of the patterning cascade model of crown morphogenesis as outlined by Jernvall [116]. Image adapted from Jernvall & Jung [131] with permission (License number: 5642721412793). © 2000 Wiley-Liss, Inc.

In an initial application of this model to Lake Ladoga seals, Jernvall [116] found the presence and size of accessory cusps to be related to height differences among earlier-forming cusps: crowns with smaller cusp height differences were more likely to develop accessory cusps (Fig 1). Seal molar cusps are aligned in a single mesiodistally oriented row, with each successive cusp added in a way that maintains this linear configuration. In this case, cusp height directly shapes the volume of the mesenchymal tissue into which activators and inhibitors diffuse (*i*.*e*., the taller the preceding cusps, the greater the basal cusp area falling outside the zone of inhibition, permitting the formation of additional cusps) [115, 116]. However, the quadrate molars of primates are characterized by much shorter, bunodont cusps and a wider occlusal surface [121]. In these teeth, the distance among early-developing cusps (and their associated zones of inhibition) are crucial determinants of accessory cusp development. Additionally, the number of cells responsible for carrying out cusp development decreases with each cusp added to the crown, leading to diminished stability in the form of later-developing cusps [115, 116, 120].

As demonstrated in comparative studies of *Tabby* mutant and wild-type mouse dentitions, the size of the developing tooth germ also plays an important role in accessory cusp morphology [101]. Tooth germs characterized by longer developmental periods will allocate more time towards overall crown growth and additional epithelial folding to create accessory cusps [122–125]. As such, teeth characterized by a relatively large crown area (*i*.*e*., molar crowns with longer developmental periods) will have a greater likelihood of developing additional cusps [126, but see 127]. While ultimate crown size is a function of several factors, including genetic predisposition [128, 129] and aspects of appositional growth rates [reviewed in 130], the temporal and spatial conditions of larger molars appear to have the most pertinent consequences for accessory cusp development.

The suitability of the PCM to explain crown variation has been tested in several taxa. Researchers found molar crown architecture to correspond to PCM expectations in Lake Ladoga seals [115, 116], mice [115], voles [115], chimpanzees [132], bonobos [132], and several human samples [127, 133–135]. However, the PCM could only partially account for tooth form variation in American black bears [136], brown bears [136], several baboon species [137], fossil hominins [138], and other human groups–of which most studies have focused on upper molar morphology [138–140].

The current study provides a novel contribution and expands on previous PCM research [127, 132, 138–140] by evaluating if model predictions align with crown configuration observed in the human lower diphyodont dentition across a relatively large sample. Here, we test for significant differences in tooth size, accessory cusp expression, and relative intercusp distance (RICD) between diphyodont metameres (deciduous lower second molars-*dm*_*2*_ and permanent lower first molars-*M*_*1*_) and permanent antimeres (permanent left-*LM*_*1*_ and right-*RM*_*1*_ first molars) (S1 Table). We expect M_1_ to be significantly larger than dm_2_, [141–143] but anticipate no significant differences in size between permanent antimeres. Any such antimeric differences would likely be due to fluctuating asymmetry [144, 145] or random measurement error [127]. Once intercusp distances are controlled for overall crown size, we expect no significant discrepancies in RICDs between diphyodont or permanent pairs. If a relationship between tooth size and accessory cusp development exists, we predict more pronounced morphological trait expression in the permanent molar compared to the deciduous molar due to the expected size differences, but no significant difference between the permanent antimeres is anticipated.

**Fig 2:**
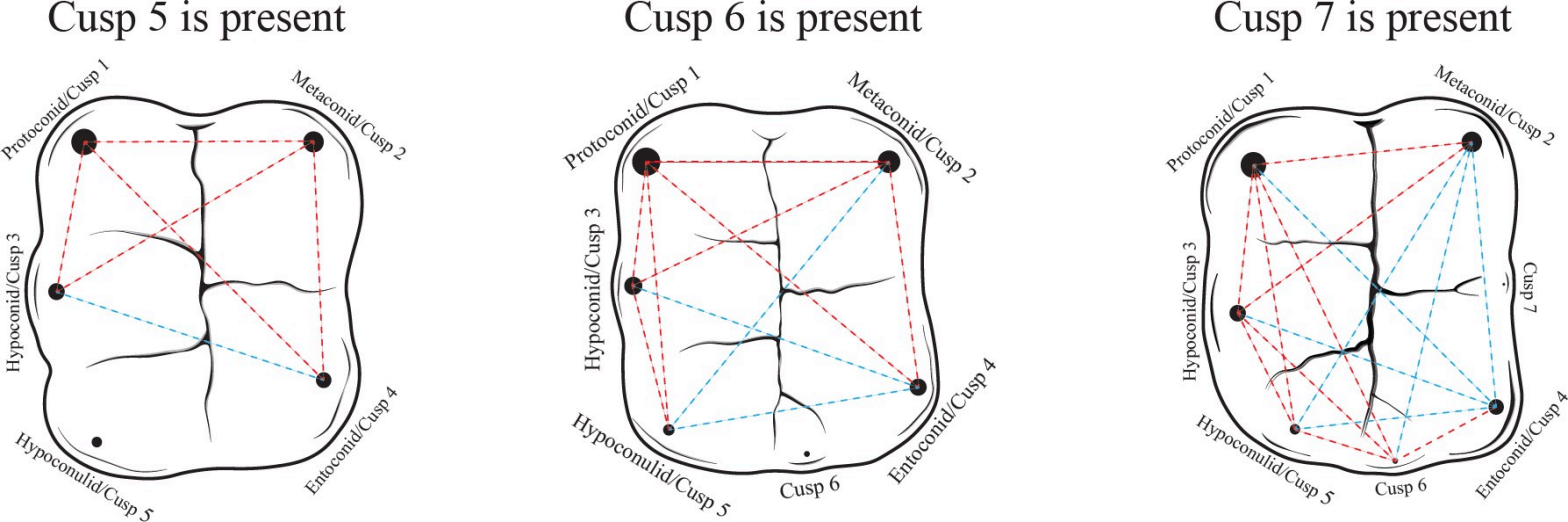
Intercusp distance expectations among early-developing cusps when a late-developing accessory cusps is present. Circles mark cusp tip position and the size of the circle corresponds to sequence of cusp development (the larger the circle, the earlier the cusp develops). Distances marked by a red dashed line indicate intercusp distances expected to be shorter when the accessory cusp is present. Distances shown with a dashed blue line illustrate intercusp distances expected to be longer when the accessory cusp is present.

Next, we investigate whether RICDs account for variation in cusp 5 (*i*.*e*., hypoconulid), cusp 6, and cusp 7 expression. PCM predictions differ slightly for accessory cusps located on the occlusal surface (*e*.*g*., cusp 5, cusp 6, cusp 7) rather than on the periphery (*e*.*g*., protosylid) of human quadrate lower molar crowns to account for the zones of inhibition surrounding primary cusps [138]. These expectations are outlined in Fig 2. Following Skinner and Gunz [132] and Ortiz and colleagues [138], we expect accessory cusps to form and exhibit higher degrees of expression if the developmental time frame of the crown is relatively long and cusp 3–4 (cusp 5), cusp 4–5 (cusp 6), and cusp 2–4 (cusp 7) distance is expanded, thus creating sufficient space outside of inhibitory fields for an accessory cusp to form. When cusp 6 is present, we expect to see an accommodating mesiobuccal shift in cusp 5 (*i*.*e*., hypoconulid), such that the cusp 2–5 distance will increase, but the cusp 1–5 and cusp 3–5 distances will decrease. When cusp 7 is present, we expect to see a distolingual shift in cusp 4, such that the cusp 1–4 and cusp 3–4 distances will increase.

## Materials and methods

For a detailed description of study methods, please refer to S1 Text. The data used in this paper were collected from 3D scans of modern human dental casts associated with an anonymized sample representing participants of the Harvard Solomon Islands Project (HSIP). The casts were collected from Solomon Islander individuals over the course of the project with participant consent. The study was deemed exempt from review by the University of Arkansas’ Office of Research Integrity and Compliance and the Institutional Review Board of Arizona State University pursuant to Federal Regulation 45CFR46(4)—Study 00007452. Loan and use of study materials were approved by the Harvard Peabody Museum and no personally identifying information was accessed as part of this research.

**Fig 3:**
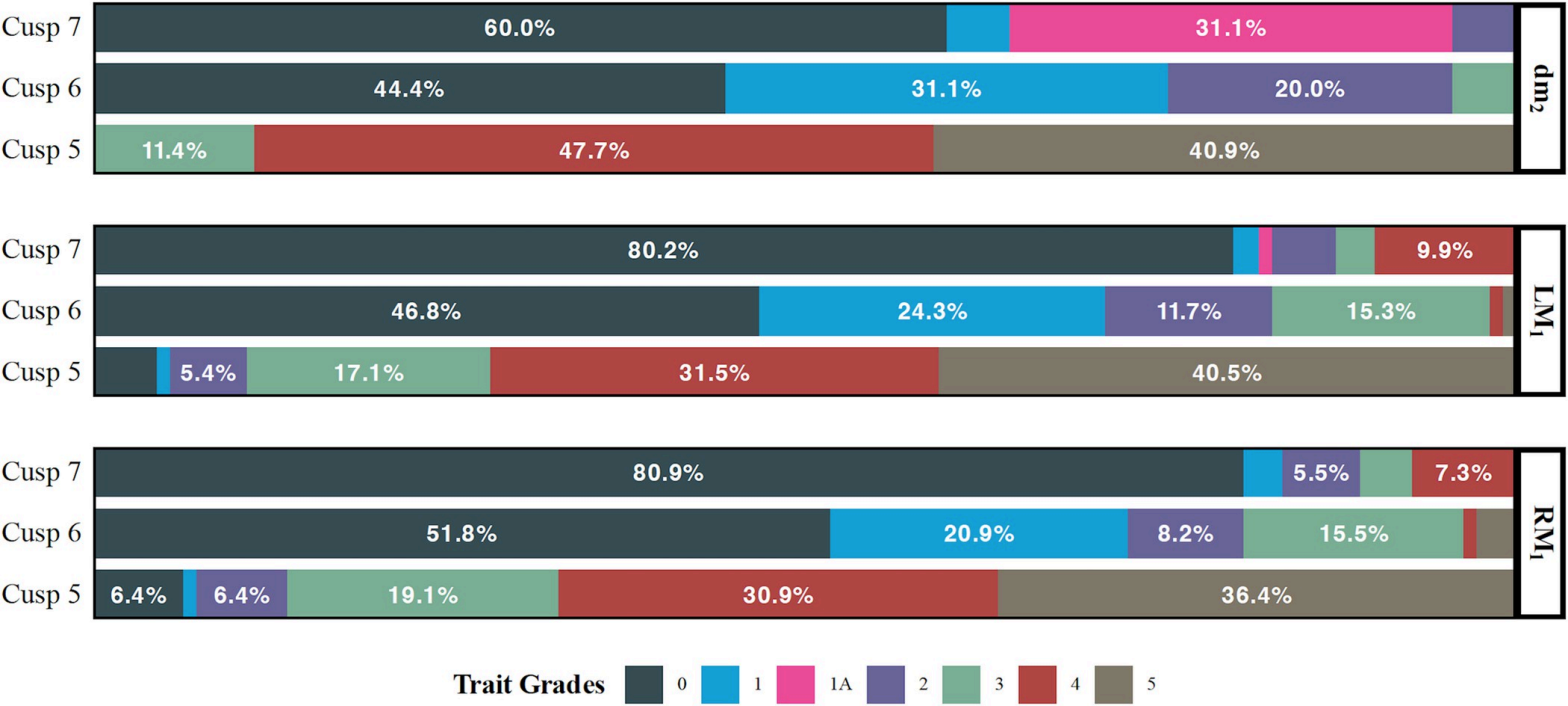
Lower molar accessory cusp frequencies in the study sample.

The study examined intercusp distance, tooth size, and morphological trait expression for 124 individuals. Tooth size dimensions were measured between margins at the maximum curvature points following established standards [146–148] (S1 Fig). Occlusal surface area was calculated by multiplying the 2D linear measurements of maximum buccolingual (BL) and mesiodistal (MD) diameter. Morphological data collection protocol followed the Arizona State University Dental Anthropology System (ASUDAS) (Fig 3; S1 Table) [148, 149].

All statistical analyses were conducted in RStudio v4.2.2 [150, 151]. Paired-samples Wilcoxon signed-rank tests were used to detect significant differences in tooth size, intercusp distance, and morphological trait expression between diphyodont metameres and permanent antimeres. Relative intercusp distances were calculated to eliminate crown size as a confounding variable by dividing the absolute intercusp distance (AICD) under consideration by the square root of the crown area (SQRTA) (*i*.*e*., RICD = AICD/SQRTA). Using the *MASS* package, proportional odds logistic regression was performed to determine if the expression of later-developing accessory cusps (*i*.*e*., cusp 5, cusp 6, and cusp 7) (dependent variable) was influenced by the relative distance between earlier-developing cusps (RICD) or occlusal surface area (a proxy for large tooth germ size and extended crown growth) as predicted by the patterning cascade model of crown morphogenesis [152]. Odds ratios were calculated to ascertain the degree to which the independent variable of interest (RICD, tooth size) impacts the odds of having an accessory cusp with more pronounced expression.

## Results

Inter- and intra-observer analyses for the morphological (S2 Table) and metric (S3 Table) data showed strong agreement both within and between observers according to guidelines provided by Landis and Koch [153] and are presented in the supplemental materials.

### Comparative analyses

The results of metameric and antimeric comparative analyses are presented in Table 1 for occlusal surface area, cusp expression, and RICD (intercusp distance scaled by occlusal surface area). Differences in occlusal surface area (p<0.000) and square root occlusal surface area (p<0.000) were significant for both metameres (dm_2_-M_1_) and permanent antimeres (LM_1_-RM_1_). This difference between metameres is due to the significantly smaller crown size characterizing deciduous as opposed to permanent molars, while antimeric differences were due to the unexpectedly larger size of right molars for this sample. When comparing morphology, only differences in cusp 5 expression reached significance for metamere (p<0.000) and antimere (p = 0.003) pairs.

**Table 1:** Results of metameric and antimeric comparative analyses for occlusal surface area, ASUDAS morphology, and RICD.

		Metameric Comparison					Antimeric Comparison				
		dm_2_ Mean	M_1_ Mean	rTEM (%)^2^	V^3^	p-value^4^	LM_1_ Mean	RM_1_ Mean	rTEM (%)	V	p-value
Occlusal Surface Area (mm^2^)	Occlusal Surface Area	**87.220**	**109.882**	**18.020**	**17**	**0.000**	**110.878**	**113.755**	**5.974**	**4012**	**0.002**
	Square Root Crown Area	**9.381**	**10.464**	**8.986**	**17**	**0.000**	**10.510**	**10.649**	**3.089**	**1980**	**0.002**
ASUDAS	Cusp 5	**4.310**	**3.310**	**33.646**	**480.5**	**0.000**	**3.927**	**3.789**	**9.123**	**28.5**	**0.003**
	Cusp 6	0.837	1.233	86.553	76	0.053	1.000	0.982	48.335	110	0.858
	Cusp 7	0.200	0.367	245.372	4	0.410	0.602	0.546	64.918	50.5	0.356
Relative Intercusp Distance (mm)^1^	Cusp 1–2	**0.481**	**0.569**	**14.886**	**43**	**0.000**	**0.564**	**0.583**	**6.689**	**4314**	**0.000**
	Cusp 1–3	**0.460**	**0.509**	**13.295**	**173**	**0.000**	0.501	0.492	8.926	2431	0.087
	Cusp 1–4	**0.864**	**0.894**	**6.170**	**174**	**0.000**	0.896	0.900	3.495	3317	0.335
	Cusp 1–5	0.858	0.826	7.963	580	0.053	0.832	0.823	4.450	2052	0.055
	Cusp 1–6	**0.997**	**0.926**	**8.932**	**142**	**0.012**	0.956	0.944	3.066	436	0.052
	Cusp 1–7	0.606	0.757	17.794	0	0.250	0.776	0.771	4.155	80	0.832
	Cusp 2–3	**0.711**	**0.823**	**13.103**	**52**	**0.000**	0.813	0.816	4.798	3177	0.588
	Cusp 2–4	**0.624**	**0.648**	**6.813**	**232**	**0.003**	0.652	0.656	5.407	3355	0.281
	Cusp 2–5	0.951	0.957	4.599	336	0.226	0.968	0.974	3.172	3133	0.091
	Cusp 2–6	0.892	0.899	5.856	62	0.325	0.906	0.911	3.339	722	0.417
	Cusp 2–7	0.283	0.353	30.426	2	0.750	**0.390**	**0.366**	**9.224**	**37**	**0.034**
	Cusp 3–4	**0.700**	**0.717**	**6.046**	**293**	**0.029**	0.718	0.718	4.407	2686	0.347
	Cusp 3–5	**0.425**	**0.366**	**17.324**	**748**	**0.000**	0.377	0.373	10.282	2386	0.423
	Cusp 3–6	**0.675**	**0.564**	**15.397**	**167**	**0.000**	**0.608**	**0.568**	**15.054**	**335**	**0.001**
	Cusp 3–7	0.639	0.739	11.664	0	0.250	0.762	0.747	7.641	80	0.832
	Cusp 4–5	**0.604**	**0.573**	**9.533**	**615**	**0.016**	0.586	0.585	6.032	2644	0.955
	Cusp 4–6	**0.347**	**0.384**	**15.360**	**39**	**0.043**	0.363	0.374	9.261	839	0.052
	Cusp 4–7	0.307	0.310	36.322	3	1.000	**0.322**	**0.357**	**14.331**	**132**	**0.043**
	Cusp 5–6	**0.402**	**0.301**	**24.093**	**168**	**0.000**	0.328	0.305	17.944	461	0.089
	Cusp 5–7	0.757	0.760	6.370	3	1.000	0.774	0.769	4.190	59	0.431
	Cusp 6–7	0.694	0.592	11.247	1	1.000	0.599	0.663	8.864	10	0.125

^1^Intercusp distance scaled by occlusal surface area

^2^rTEM: Relative technical error of measurement

^3^V: Paired-samples Wilcoxon signed-rank test statistic

^4^α≤0.050; significant results in bold

The examination of metameric differences in paired RICDs all yielded significant results, except distances originating from cusp 7 and the distance between cusps 1–5 (p = 0.053), cusps 2–5 (p = 0.226), and cusps 2–6 (p = 0.325). The majority of these disparities stemmed from larger RICDs in permanent molars, apart from distances involving cusps 5 and 6, which were generally longer in deciduous molars. Considering permanent antimeres, only cusp 1–2 (p<0.000), cusp 2–7 (p = 0.034), cusp 3–6 (p = 0.001), and cusp 4–7 (p = 0.043) spacing differed significantly. Since there were no consistent patterns to the size discrepancies pointing to a wider configuration in one of the antimeres, random error or fluctuating asymmetry was likely responsible for these findings. The results involving cusp 7 should be interpreted with caution as the sample of dentitions expressing cusp 7 is small (dm_2_ n = 4; M_1_ n = 5).

### Regression analyses

We first tested the PCM assumption that larger crown size, reflecting an extended period of development, increases the odds of having accessory lower molar cusps with higher trait expression. Tooth size was not a significant predictor of cusp 5 (p = 0.817), cusp 6 (p = 0.303), or cusp 7 (p = 0.157) expression in dm_2_. In permanent molars, tooth size was a significant predictor of cusp 5 expression for the left (p = 0.031) and the right (p = 0.007) antimere, as well as cusp 6 (p = 0.048) expression in RM_1_. These results were driven by effects in the expected direction, larger crown size increasing the odds of greater accessory cusp expression in these teeth. However, the current findings may be influenced by sex-specific differences in tooth dimensions and/or morphological trait expression.

**Fig 4:**
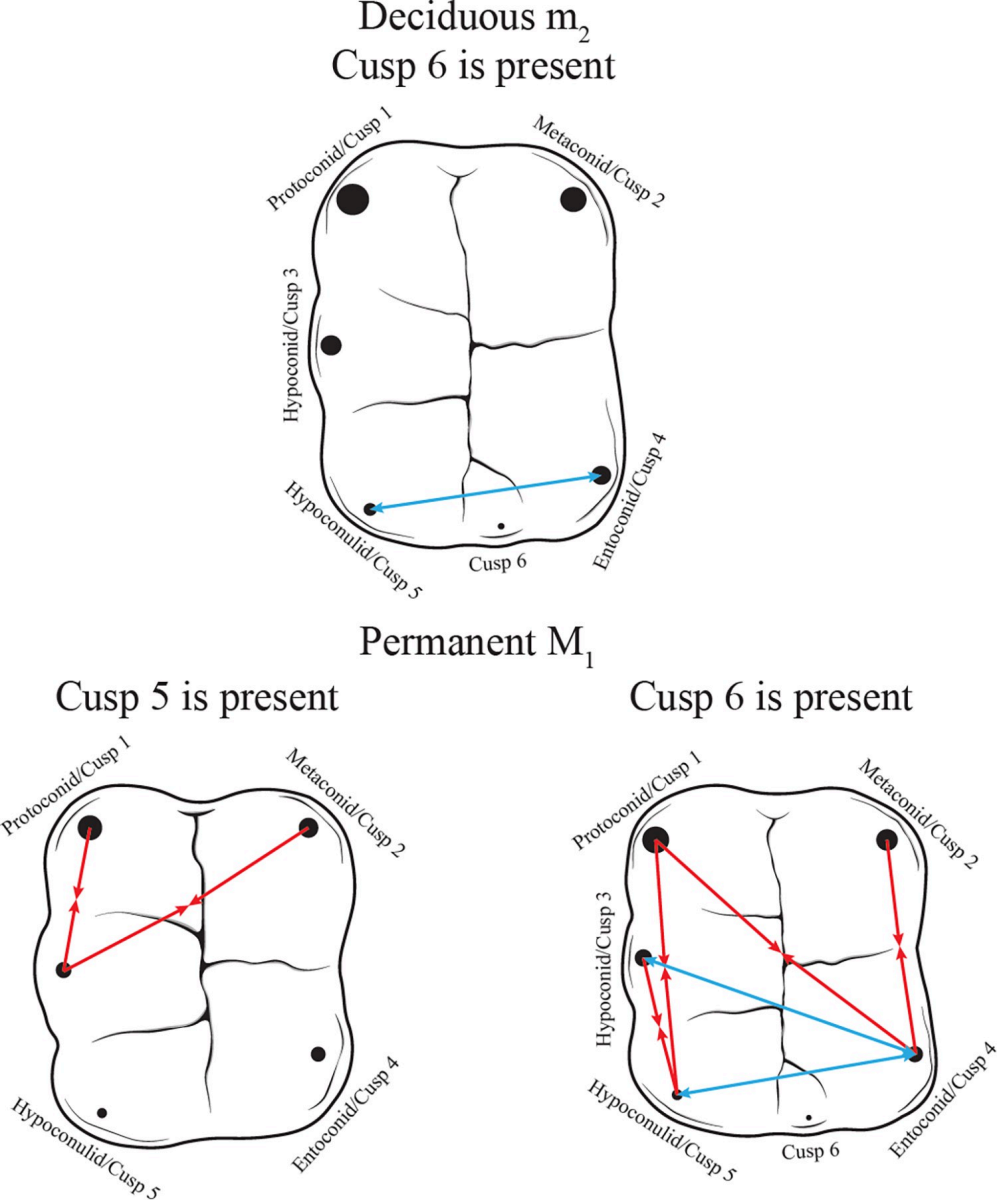
Relative intercusp distances that play an important role in shaping accessory cusp expression in deciduous molars and both permanent antimeres. Red arrows pointing toward each other indicate a negative relationship between intercusp distance and morphological trait expression. Blue arrows pointing away from each other signal a positive relationship between intercusp distance and morphological trait expression.

We next examined RICD as a predictor of accessory cusp expression, scaling the intercusp distances by occlusal surface area to eliminate tooth size as a confounding factor in the analyses (Table 2; Fig 4). For dm_2_, the only significant predictor of accessory cusp expression was the distance between cusps 4 and 5 (p<0.000), those with relatively greater intercusp spacing having almost 10 times higher odds of exhibiting a larger cusp 6 for every 0.10 unit increase in intercusp distance. For both permanent antimeres, an increase in cusp 1–3 and cusp 2–3 distances lowered the odds of having a cusp 5 with higher expression. In LM_1_, the odds of having a larger cusp 5 decreased by 68% and 64% for every 0.10 unit increase in the cusp 1–3 (p<0.000) and cusp 2–3 (p<0.000) distances, respectively. For RM_1_, the odds of greater cusp 5 expression diminished by 75% and 60% as cusp the 1–3 (p<0.000) and cusp 2–3 (p<0.000) distances expanded, respectively.

**Table 2:** Results of proportional odds regression when using occlusal surface area and RICD scaled by occlusal surface area as a predictor of lower molar accessory cusp trait expression. Cells highlighted in blue represent intercusp distances that are significantly longer in teeth with more pronounced accessory cusp expression. Cells highlighted in red represent intercusp distances that are significantly shorter in molars characterized by greater accessory cusp expression.

			OSA^4^	Cusp 1–2	Cusp 1–3	Cusp 1–4	Cusp 1–5	Cusp 1–6	Cusp 2–3	Cusp 2–4	Cusp 2–5	Cusp 2–6	Cusp 3–4	Cusp 3–5	Cusp 3–6	Cusp 4–5	Cusp 4–6	Cusp 5–6
dm_2_	Cusp 5	Coefficient	0.008	3.212	-3.764*	3.445*	-	-	-1.180	3.378	-	-	1.3299	-	-	-	-	-
		LRχ^1^	0.053	0.439	0.664	0.845	-	-	0.090	0.818	-	-	0.065	-	-	-	-	-
		p-value^2^	0.817	0.508	0.415	0.358	-	-	0.764	0.366	-	-	0.799	-	-	-	-	-
		Odds ratio^3^	-	-	-	-	-	-	-	-	-	-	-	-	-	-	-	-
	Cusp 6	Coefficient	0.034	-4.531	2.075	-2.920	-0.640	-	0.712	-5.275	3.144	-	4.372	-9.895*	-	**22.666** *	-	-
		LRχ	1.061	0.734	0.207	0.552	0.022	-	0.033	1.728	0.420	-	0.662	1.857	-	**13.670**	-	-
		p-value	0.303	0.392	0.649	0.458	0.883	-	0.856	0.189	0.517	-	0.416	0.173	-	**0.000**	-	-
		Odds ratio	-	-	-	-	-	-	-	-	-	-	-	-	-	**9.646**	-	-
	Cusp 7	Coefficient	0.129	-7.393	-4.500	-6.648	-16.176	-13.828*	-3.152	-0.898	-5.365	-10.666	-9.553	-31.484	-8.960	1.380	-29.151	2.659
		LRχ	2.000	0.517	0.268	0.699	2.187	1.533	0.184	0.017	0.330	0.593	0.856	3.433	0.354	0.021	3.202	0.054
		p-value	0.157	0.472	0.605	0.403	0.139	0.216	0.668	0.896	0.566	0.441	0.355	0.064	0.552	0.886	0.074	0.816
		Odds ratio	-	-	-	-	-	-	-	-	-	-	-	-	-	-	-	-
LM_1_	Cusp 5	Coefficient	**0.030**	-2.528	**-11.374** *	-1.123*	-	-	**-10.137**	-1.787	-	-	1.224	-	-	-	-	-
		LRχ	**4.678**	0.654	**16.680**	0.181	-	-	**13.519**	0.630	-	-	0.146	-	-	-	-	-
		p-value	**0.031**	0.419	**0.000**	0.670	-	-	**0.000**	0.428	-	-	0.703	-	-	-	-	-
		Odds ratio	**1.003**	-	**0.321**	-	-	-	**0.363**	-	-	-	-	-	-	-	-	-
	Cusp 6	Coefficient	0.022	-3.551	-3.535	**-7.437**	**-5.903**	**-**	-0.071	**-7.177**	1.958	**-**	**8.130**	**-7.289**	**-**	**18.072**	-	-
		LRχ	2.720	1.161	1.870	**7.035**	**4.217**	**-**	0.001	**8.943**	0.405	**-**	**5.442**	**4.794**	**-**	**29.570**	-	-
		p-value	0.099	0.281	0.171	**0.008**	**0.040**	**-**	0.978	**0.003**	0.525	**-**	**0.020**	**0.029**	**-**	**0.000**	-	-
		Odds ratio	-	-	-	**0.475**	**0.554**	-	-	**0.488**	-	-	**2.255**	**0.482**	-	**6.093**	-	-
	Cusp 7	Coefficient	0.022	-4.455	-5.184	-1.248	-3.679	-13.122	**-6.906**	3.204	-4.972	-7.766	-8.212*	-1.867	-13.502	-6.122*	-5.283	2.981
		LRχ	1.424	1.043	2.134	0.130	0.975	2.979	**4.265**	1.050	1.544	1.876	3.769	0.201	3.472	3.446	0.662	0.158
		p-value	0.233	0.307	0.144	0.718	0.324	0.084	**0.039**	0.305	0.214	0.171	0.052	0.654	0.062	0.063	0.416	0.691
		Odds ratio	-	-	-	-	-	-	**0.501**	-	-	-	-	-	-	-	-	-
RM_1_	Cusp 5	Coefficient	**0.039**	-1.324	**-13.893** *	-0.215	-	-	**-9.200**	1.373	-	-	2.558	-	-	-	-	-
		LRχ	**7.159**	0.125	**21.433**	0.001	-	-	**8.349**	0.362	-	-	0.513	-	-	-	-	-
		p-value	**0.007**	0.724	**0.000**	0.938	-	-	**0.004**	0.547	-	-	0.474	-	-	-	-	-
		Odds ratio	**1.004**	-	**0.249**	-	-	-	**0.399**	-	-	-	-	-	-	-	-	-
	Cusp 6	Coefficient	**0.028**	**-7.885**	-3.718	**-7.147**	**-6.821** *	-	0.023	**-7.952**	1.974*	-	**13.126**	**-11.969**	-	**23.616**	-	-
		LRχ	**3.894**	**4.251**	1.725	**6.105**	**4.693**	-	0.000	**9.895**	0.343	-	**11.333**	**9.368**	-	**43.473**	-	-
		p-value	**0.048**	**0.039**	0.189	**0.013**	**0.030**	-	0.994	**0.002**	0.558	-	**0.001**	**0.002**	-	**0.000**	-	-
		Odds ratio	**1.003**	**0.455**	-	**0.489**	**0.506**	-	-	**0.452**	-	-	**3.716**	**0.302**	-	**10.608**	-	-
	Cusp 7	Coefficient	0.032	-3.832	-5.073	0.500	-0.258	-1.447	-7.824	5.890	-5.415	-0.386	**-11.688**	5.051	-2.928	**-16.586**	-8.491	-4.693
		LRχ	2.751	0.628	1.587	0.019	0.004	0.032	3.623	2.909	1.673	0.002	**5.436**	1.077	1.494	**14.883**	1.538	1.395
		p-value	0.097	0.428	0.208	0.890	0.948	0.859	0.057	0.088	0.196	0.962	**0.020**	0.300	0.222	**0.000**	0.215	0.238
		Odds ratio	-	-	-	-	-	-	-	-	-	-	**0.311**	-	-	**0.190**	-	-

*Values violating the assumption of proportionality are marked with an asterisk, these results should be interpreted with caution

^1^LRχ: Likelihood ratio chi-square test statistic

^2^α≤0.050; significant results in bold

^3^Odds ratio obtained by dividing the model coefficient by 10 to scale the odds ratio to a 0.10-unit change and exponentiating the quotient

^4^OSA: Occlusal surface area (OSA = BLxMD)

Numerous intercusp distances played an important role in permanent molar cusp 6 expression. For both antimeres, an increase in the cusp 1–4, cusp 1–5, cusp 2–4, and cusp 3–5 distances lowered the odds of presenting with a larger cusp 6. A longer distance between the cusps surrounding cusp 6 boosted the odds of having a larger accessory cusp. On the right side, the odds of more pronounced cusp 6 expression rose approximately four-fold (p = 0.001) and 10-fold (p<0.000), while on the left, two times (p = 0.020) and six times (p<0.000) higher odds were observable with each 0.10 unit increase in the cusp 3–4 and cusp 4–5 distances, respectively. These results are consistent with our original prediction that having a cusp 6 would cause a mesiobuccal shift in the hypoconulid as evidenced by the significant role of the diminished cusp 1–5 and cusp 3–5 spacings when cusp 6 is present. Additionally, the mesiolingual translocation of cusp 4 (*i*.*e*., entoconid) appears to be just as pertinent to make space for cusp 6. This is supported by the importance an expansion in the cusp 3–4 and cusp 4–5 distances, signaling lingual drift, and a reduction in the cusp 1–4 and cusp 2–4 distances, consistent with mesial movement. The predicted relationship between accessory cusp expression and cusp 2–5 spacing was also observable, though it did not reach the level of significance as was initially expected.

Cusp 7 expression was shaped by the distance between cusps 2 and 3 (p = 0.039) on the left side. The odds of displaying a larger cusp 7 diminished by 50% for every added 0.10 unit in this measurement. On the right side, a reduction of the cusp 3–4 (p = 0.020) and cusp 4–5 (p<0.000) distances was an important predictor of cusp 7 expression. For every 0.10 unit increase in the cusp 3–4 and cusp 4–5 spacings, the odds of having a cusp 7 with more pronounced expression were 69% and 81% lower, respectively. The role of shorter cusp 3–4 and cusp 4–5 distances seen in the right permanent antimere indicated a mesiobuccal, rather than a distolingual, shift in cusp 4, contrary to original expectations. While the same pattern can be observed in the left antimere, these variables only reached marginal significance. Interestingly, the predicted distance increase between cusps 2 and 4 to accommodate the accessory cusp wedged between these primary cusps was not a significant predictor of trait expression on either side of the dentition.

## Discussion

To our knowledge, this study is the first to utilize a large sample of contemporary human dentitions to examine morphological variation of deciduous and permanent lower molars within a PCM framework. The configuration of lower molars is distinct from those of upper molars, and the question of how PCM-derived expectations are applied to lower molar accessory cusps, which are more centrally located, has been broached by previous studies (*e*.*g*., 132, 138), but to the exclusion of deciduous elements. As such, these findings mark a novel contribution and provide insight into the foundations of mandibular molar variation.

### Comparative analyses

This study identified significant differences in tooth size and cusp 5 expression between deciduous and permanent metameres, corroborating previous findings [139]. The significant difference in metameric cusp 5 expression may be due to disparate susceptibility to environmental influences characterizing the deciduous and permanent dentitions. The dm_2_ almost always exhibited a cusp 5, while expression on M_1_s tended to be more variable. This finding may point to greater canalization of the deciduous molar form and less susceptibility to environmental influence compared to its permanent counterpart [27, 154, 155], though recent diphyodont metamere heritability comparisons failed to find support for this hypothesis [156]. Significant discrepancies in cusp 5 expression between antimeres may also support the notion that this cusp is susceptible to environmental influences, consistent with previous findings [145, 157]. Non-significant differences in metameric and antimeric cusp 6 expression are compatible with previous research, but the lack of difference in cusp 7 expression between tooth pairs differs from other studies, which identified more pronounced cusp 7 expression in dm_2_ [158, 159]. The lack of significant difference in metameric and antimeric cusp 6 and cusp 7 expression suggests these accessory cusps are more resilient to environmental disturbance than cusp 5. This rationale is bolstered by heritability studies, which have found cusp 6 and cusp 7 to be characterized by a strong additive genetic component [156, 160, 161].

Paired tests indicate that most RICDs significantly differ between metameres. For distances involving cusp 7, inconsistent results for the antimeric and metameric comparisons may relate to the relatively small number of individuals that possess this accessory cusp within the study sample. Differences between the other RICDs may reflect the distinct crown configurations associated with deciduous and permanent elements, in particular, significant discrepancies in cusp 5 expression (see Table 1). The reason for significantly different cusp 1–2 and cusp 3–6 distances between the permanent antimeres is also unclear. It is possible, however, that because these dimensions involved the earliest-forming cusps, they have the greatest potential for variation.

### Regression analyses

An important aim of this study was to establish if tooth size and RICDs were important predictors of accessory cusp formation. This study is the first to test PCM expectations in lower dm_2_, though previous work has explored this model and its associated predictions for Carabelli’s trait in dm^2^ [139]. Neither tooth size nor RICD seemed to be a predictor of deciduous lower molar accessory cusp expression. Only the distance between cusps 4 and 5 was found to play a significant role in cusp 6 expression, which is consistent with PCM expectations and results obtained in permanent molars [132, 138]. The overall absence of a relationship between dm_2_ RICD, tooth size, and accessory cusp expression differs from the findings of Paul and colleagues [139], who identified more consistent relationships between RICDs and accessory cusp expression in the deciduous rather than permanent dentition. The current results may diverge from those of Paul and colleagues [139] due to the lack of variation exhibited by cusp 5 and the limited number of individuals displaying a cusp 7. Another plausible explanation may be the differing cusp configurations that characterize maxillary and mandibular molars. The peripheral location of Carabelli’s trait may correspond to PCM expectations more closely than the occlusally located accessory cusps examined in this study. Since no other study has tested PCM predictions in dm_2_, further research with larger sample sizes, especially those characterized by a wider range of morphological variation from diverse populations, would help contextualize these results. Nevertheless, the ability of the current study to highlight the importance of cusp 4–5 distance in predicting cusp 6 expression indicates that cusp 6 is a true accessory cusp with a developmental pattern closely shaped by the spatial relationship among earlier-developing cusps.

Size-related variables were significant predictors of accessory cusp expression in permanent molars. These results align with those of previous work that identified a link between larger tooth size and increased crown complexity [122, 124, 125, 132, 162]. In particular, Dahlberg [122] and Garn and colleagues [162] found longer M_1_s to have a greater likelihood of exhibiting five cusps instead of four. Skinner and Gunz [132] also discovered that members of the genus *Pan* with a cusp 6 had significantly larger teeth. Expansion of the crown’s buccolingual dimension could increase the likelihood of areas along the mesial and distal marginal ridges falling outside the inhibition zones of primary cusps, thus promoting accessory cusp development in accordance with the PCM.

For permanent antimeres, a reduction in cusp 1–3 and cusp 2–3 spacings was associated with having a larger cusp 5. These findings are consistent with PCM expectations, whereby a mesiolingual shift in cusp 3 (*i*.*e*., hypoconid) would serve to provide adequate space for a buccally-skewed cusp 5. While an increased cusp 3–4 distance was observed in both antimeres in concordance with the PCM, none of these variables reached the level of significance. The lack of such a relationship in the current study departs from the findings of Ortiz and colleagues [138], who observed a significant increase in this distance with greater cusp 5 expression in both fossil hominins and recent modern humans. This study’s failure to replicate previous results may relate to divergent sample composition. It is also possible that a transverse expansion of the talonid is not strictly necessary to accommodate a cusp 5 since cusps 3 and 4 may already be sufficiently distant in a four-cusped molar.

Several RICD variables emerged as important predictors of cusp 6 expression. Consistent with our initial predictions, the results of the proportional odds logistic regression indicated a mesiobuccal shift in cusp 5 (*i*.*e*., hypoconulid) to integrate cusp 6. However, the mesiobuccal shift of cusp 4 (*i*.*e*., entoconid) was also revealed by shorter cusp 1–4 and cusp 2–4 distances in individuals with more pronounced cusp 6 expression. This mesiobuccal drift seen in the entoconid is significant enough that the cusp 1–4 and cusp 2–4 distances were reduced, but not excessively, as increased cusp 3–4 and 4–5 spacings predicted greater cusp 6 expression. Supporting the current study’s original hypothesis, an increased cusp 4–5 distance significantly shaped cusp 6 expression. This relationship appears to be a crucial determinant of trait manifestation since its role in cusp 6 expression has been documented in the *Pan*, *Pongo*, *Australopithecus*, *Paranthropus*, and *Homo* genera as well as another recent modern human sample [132, 138]. The consistent influence of this predictor across studies suggests that while a reduction in intercusp distances may be important for greater accessory cusp expression, broadening the gap between certain cusps to expose regions of the crown outside of inhibition zones could be equally necessary. This is not explicitly outlined in the original model [116], but was posited by previous scholars who investigated the evolution of lower molar crown configuration [132, 138]. The importance of expanded distances between certain earlier developing cusps is more than likely related to the integrated cuspal arrangement characterizing the quadrate molars of primates. The accessory cusps examined in this study are more centrally located on the tooth crown as opposed to occupying a peripheral position–something more typical of accessory cusps on Lake Ladoga seal molars.

The antagonistic relationship between cusp 5 and cusp 6 expression revealed by quantitative genetic studies may also explain why these accessory cusps follow PCM expectations to variable degrees. Stojanowski and colleagues [157] and Paul and colleagues [163] uncovered negative genetic correlations between cusp 5 and cusp 6 in lower molars, indicating that these cusps may be competing for “cellular real estate” during development and the amount of resources allotted to one will have direct and opposing consequences for the development and expression of the other. Together, these findings suggest that while cusp 6 expression may follow PCM expectations closely, the presence of this cusp will cause more variability in cusp 5 size, likely leading to deviations in the degree to which this cusp conforms to model expectations.

This study was unable to identify a significant connection between increased cusp 2–4 spacing and cusp 7 expression as was hypothesized prior to analysis. The inability to identify a consistent link between RICD and cusp 7 development is not unique to the current project. Ortiz and colleagues [138] were also unable to detect such a relationship in the *Australopithecus*, *Homo*, and *Pan* genera. Kozitzky [137] sought to determine if the expression of the median lingual notch cuspule (MLNC) in the upper molars of several baboon groups followed PCM predictions. Based on its location, the MLNC may be an isomeric structure in the upper molars of *Cercopithecoidea* that is similar to cusp 7, although its spatial placement indicates a much closer association with the cingulum compared to cusp 7, which is more closely integrated within the crown. In a pooled sample, cusp 2–4 distance was not a significant predictor of MLNC expression, but when considering only the first molar, this variable was significant in hamadryas baboons [137]. The failure of cusp 7 expression to follow PCM expectations across several studies suggests a different developmental pathway may be responsible for modulating cusp 7 expression [164]. Since the overall size variation seen in cusp 7 is minimal, it is also possible that the trait expression pattern may be difficult to detect on the outer enamel surface versus the enamel-dentine junction due to enamel thickness or the presence of a corresponding dentine horn. This variation can lead to conflation of the “metaconulid-type” and “interconulid-type” cusp 7 in analyses that rely on trait expression of the external crown surface exclusively [164–168].

In sum, the current study found partial support for the PCM in dm_2_ and M_1_. Similar to previous studies, cusp 6 was consistently the accessory cusp with variables most closely following PCM expectations [132, 138]. Human upper molars have received more attention in PCM studies, with some outlining cusp patterns aligning with the PCM [117, 127, 132–135] and others only identifying partial agreement with the model [138–140]. The inability of the model to consistently account for postcanine cusp development, size, and arrangement potentially indicates that this model alone may not be sufficient to explain the morphological diversity characterizing the human dentition. However, we acknowledge that our understanding of how the model applies to human molars is currently incomplete. It is possible that certain organisms with less derived dental patterns, like Lake Ladoga seals on which the model was developed, conform more closely to PCM expectations due to greater canalization in tooth form and less susceptibility to external factors shaping development. While Salazar-Ciudad and Jernvall [114] were able to reconstruct the general quadritubercular form that resembles human upper molars through PCM simulations, they acknowledged that model parameters (*e*.*g*., epithelial growth rate, rate of epithelial activator secretion) can be modified by population variation and environmental components, though the degree to which crown form can deviate from expected patterns as a result of these factors is not well-understood. The impact of environmental variables on cusp morphogenesis is relatively understudied, but recent research suggests nutritional deprivation and subsistence strategy can alter accessory trait expression, crown size, and intercusp spacing [169]. Following Salazar-Ciudad and Jernvall [114], it is possible that environmental factors may be the reason for inconsistencies. Additional research expanding the sample composition (*e*.*g*., incorporating populations of diverse bioregional affiliation), the variables examined (*e*.*g*., deciduous and permanent antimeres, metameres, and isomeres), and accounting for various environmental factors (*e*.*g*., intrauterine environment, nutritional deprivation, pathological conditions, physiological stress) in future studies will shed light on how and to what extent these elements alter morphological compatibility with PCM predictions.

Understanding the developmental foundations of dental variation is key to fine-tuning our use of dental phenotypes for reconstructing human evolutionary history. Indeed, insights into the developmental regulation of cuspal patterning can help us to probe the evolution of crown form. For example, identifying characters prone to homoplasy can help us to improve phylogenetic methods (167). Although outside the scope of the current study, we hope researchers can leverage current findings to approach specific questions surrounding macroevolution of crown form in the hominoid fossil record or microevolutionary changes characterizing contemporary populations [170–173].

## Supporting information

S1 Fig Visual illustration of the data collection methodology followed by the current study. Red circles mark the cusp tip location. The horizontal red line at the top of the image shows the linear size reference tool used to scale all measurements. The blue perpendicular lines indicate the mesiodistal and buccolingual tooth dimensions. Image courtesy of senior author’s (KSP) personal collection.
(TIFF)

S1 Table Grading system used to assess accessory cusp expression and grade frequencies for study sample. (DOCX)

S2 Table Cohen’s weighted kappa (κ) indicating observer agreement for morphological traits. (DOCX)

S3 Table Different measures of intra-observer error for absolute intercusp distance measurements. (DOCX)

S4 Table Complete metric and morphological dataset utilized in study. (XLSX)

S1 Text Extended description of materials and methods. (DOCX)
