# Supplementary figures and images for: Evaluating predictions of the patterning cascade model of crown morphogenesis in the human lower mixed and permanent dentition
Source: PLoS One. 2024 Jun 27;19(6):e0304455. doi: 10.1371/journal.pone.0304455 (PMC11210800; doi:10.1371/journal.pone.0304455)

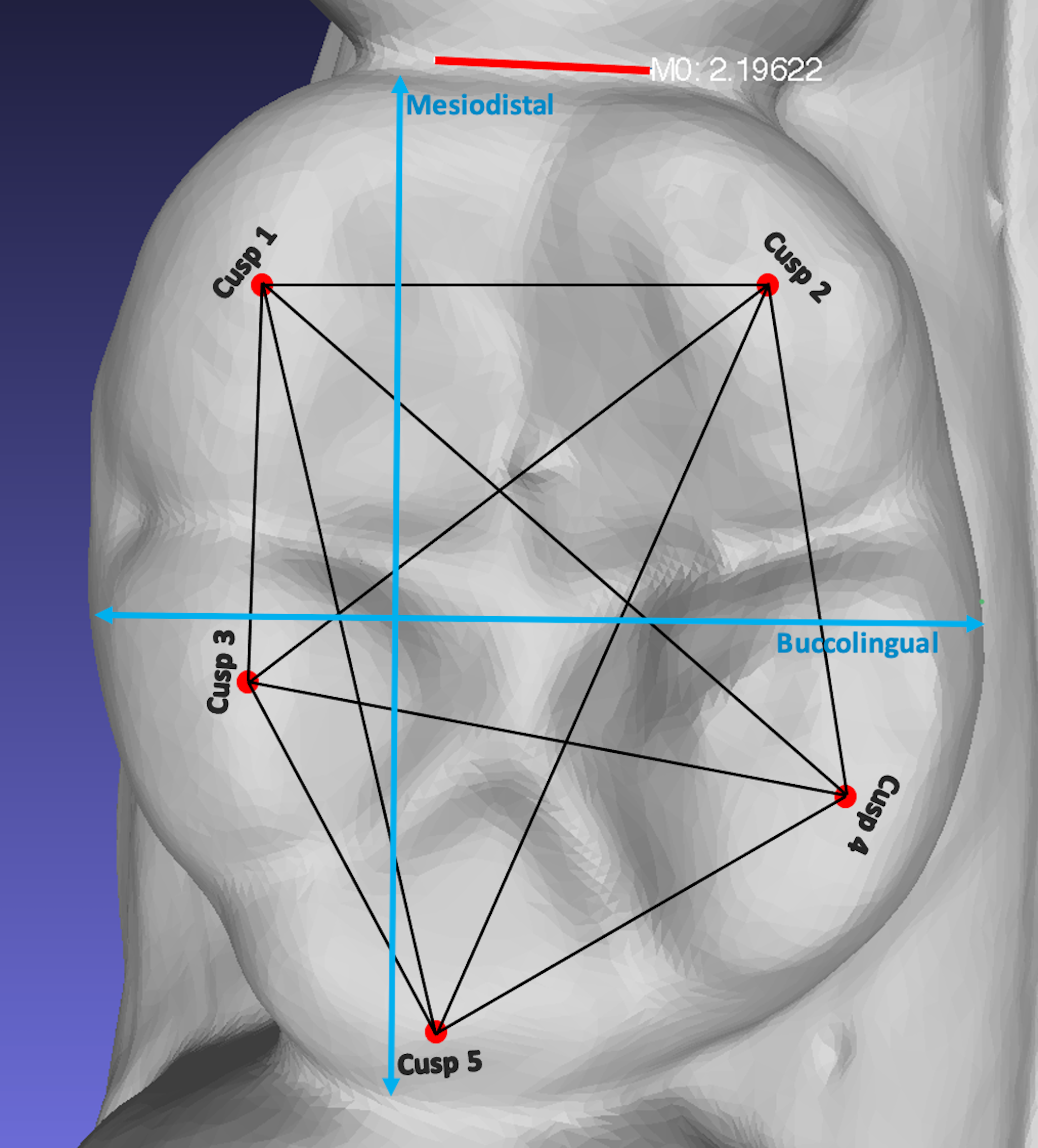

Supplement: S1 Fig — Red circles mark the cusp tip location. The horizontal red line at the top of the image shows the linear size reference tool used to scale all measurements. The blue perpendicular lines indicate the mesiodistal and buccolingual tooth dimensions. Image courtesy of senior author’s (KSP) personal collection. (TIFF) [file pone.0304455.s001.tiff]
