# Supplementary material for: Evaluating predictions of the patterning cascade model of crown morphogenesis in the human lower mixed and permanent dentition
Source: PLoS One. 2024 Jun 27;19(6):e0304455. doi: 10.1371/journal.pone.0304455 (PMC11210800; doi:10.1371/journal.pone.0304455)
Supplement: S1 Table — (DOCX) [file pone.0304455.s002.docx]

**S1 Table. Grading system used to assess accessory cusp expression following Scott & Irish [149] and grade frequencies for study sample.**

| Trait | Grade | Definition | dm_2_ | | LM_1_ | | RM_1_ | |
| --- | --- | --- | --- | --- | --- | --- | --- | --- |
|  |  |  | % | n | % | n | % | n |
| Cusp 5  (Hypoconulid) | 0 | Hypoconulid is absent (four-cusped tooth) | 0.00 | 0/44 | 4.50 | 5/111 | 6.36 | 7/110 |
|  | 1 | Trace expression | 0.00 | 0/44 | 0.90 | 1/111 | 0.09 | 1/110 |
|  | 2 | Slight | 0.00 | 0/44 | 5.40 | 6/111 | 6.36 | 7/110 |
|  | 3 | Moderate | 11.4 | 5/44 | 17.1 | 19/111 | 19.1 | 21/110 |
|  | 4 | Strong | 47.7 | 21/44 | 31.5 | 35/111 | 30.9 | 34/110 |
|  | 5 | Pronounced | 40.9 | 18/44 | 40.5 | 45/111 | 36.4 | 40/110 |
| Cusp 6  (*Tuberculum sextum*) | 0 | Absence of cusp 6 | 44.4 | 20/45 | 46.8 | 52/111 | 51.8 | 57/110 |
|  | 1 | Cusp 5 is more than twice the size of cusp 6 | 31.1 | 14/45 | 24.3 | 27/111 | 20.9 | 23/110 |
|  | 2 | Cusp 5 is about twice the size of cusp 6 | 20.0 | 9/45 | 11.7 | 13/111 | 8.18 | 9/110 |
|  | 3 | Cusp 5 and cusp 6 are about equal in size | 4.44 | 2/45 | 15.3 | 17/111 | 15.5 | 17/110 |
|  | 4 | Cusp 6 is slightly larger than cusp 5 | 0.00 | 0/45 | 0.09 | 1/111 | 0.09 | 1/110 |
|  | 5 | Cusp 6 is markedly larger than cusp 5 | 0.00 | 0/45 | 0.09 | 1/111 | 2.73 | 3/110 |
| Cusp 7  (*Tuberculum intermedium*) | 0 | No accessory cusp between cusps 2 and 4 | 60.0 | 27/45 | 80.2 | 89/111 | 80.9 | 89/110 |
|  | 1A | This expression does not assume the typical wedge-shaped form of a cusp 7, but is marked by a groove on the lingual surface of the metaconid | 31.1 | 14/45 | 0.09 | 1/111 | 0.00 | 0/110 |
|  | 1 | Small, wedge-shaped cusp between cusps 2 and 4 | 4.44 | 2/45 | 1.80 | 2/111 | 2.73 | 3/110 |
|  | 2 | Distinct, but small cusp | 4.44 | 2/45 | 4.50 | 5/111 | 5.45 | 6/110 |
|  | 3 | Moderate cusp | 0.00 | 0/45 | 2.70 | 3/111 | 3.64 | 4/110 |
|  | 4 | Large cusp | 0.00 | 0/45 | 9.91 | 11/111 | 7.27 | 8/110 |
