# Supplementary material for: Evaluating predictions of the patterning cascade model of crown morphogenesis in the human lower mixed and permanent dentition
Source: PLoS One. 2024 Jun 27;19(6):e0304455. doi: 10.1371/journal.pone.0304455 (PMC11210800; doi:10.1371/journal.pone.0304455)
Supplement: S2 Table — (DOCX) [file pone.0304455.s003.docx]

**S2 Table. Cohen’s weighted kappa (κ) indicating observer agreement for morphological traits.**

|  | Intraobserver error | | | Interobserver error | | |
| --- | --- | --- | --- | --- | --- | --- |
|  | Cusp 5 | Cusp 6 | Cusp 7 | Cusp 5 | Cusp 6 | Cusp 7 |
| LM_1_ | 0.913 | 0.958 | 0.853 | 0.865 | 0.866 | 0.856 |
| RM_1_ | 0.96 | 0.91 | 0.893 | 0.802 | 0.785 | 0.674 |
