# Supplementary material for: Evaluating predictions of the patterning cascade model of crown morphogenesis in the human lower mixed and permanent dentition
Source: PLoS One. 2024 Jun 27;19(6):e0304455. doi: 10.1371/journal.pone.0304455 (PMC11210800; doi:10.1371/journal.pone.0304455)
Supplement: S3 Table — (DOCX) [file pone.0304455.s004.docx]

**S3 Table. Different measures of intra-observer error for absolute intercusp distance measurements.**

|  | dm_2_ | | | | | LM_1_ | | | | | RM_1_ | | | | | |
| --- | --- | --- | --- | --- | --- | --- | --- | --- | --- | --- | --- | --- | --- | --- | --- | --- |
|  | MAE | MAPE | RMSE | TEM | rTEM | MAE | MAPE | RMSE | TEM | rTEM | MAE | MAPE | RMSE | TEM | rTEM |  |
| Cusp 1-2 | 0.3139333 | 7.10% | 0.3681528 | 0.2603233 | 5.68% | 0.3875333 | 6.73% | 0.4341853 | 0.3070154 | 5.17% | 0.3854667 | 6.38% | 0.4881221 | 0.34515450 | 5.74% |  |
| Cusp 1-3 | 0.2672667 | 6.38% | 0.3187249 | 0.2253725 | 5.35% | 0.5300000 | 10.37% | 0.6163899 | 0.4358535 | 7.99% | 0.3276667 | 6.59% | 0.4005374 | 0.28322270 | 5.40% |  |
| Cusp 1-4 | 0.4729333 | 6.03% | 0.6026081 | 0.4261083 | 5.23% | 0.5196667 | 5.67% | 0.6527354 | 0.4615536 | 4.88% | 0.4068667 | 4.58% | 0.4919395 | 0.34785380 | 3.71% |  |
| Cusp 1-5 | 0.3966667 | 5.06% | 0.5282853 | 0.3735541 | 4.61% | 0.6843846 | 8.01% | 0.7882435 | 0.5573723 | 6.34% | 0.4507500 | 5.35% | 0.4981901 | 0.35227360 | 4.08% |  |
| Cusp 1-6 | 0.2147500 | 2.34% | 0.3153213 | 0.2229658 | 2.37% | 0.8515000 | 9.21% | 0.9422196 | 0.6662499 | 6.86% | 0.4504000 | 4.61% | 0.4914538 | 0.34751030 | 3.49% |  |
| Cusp 1-7 | 0.4110000 | 6.45% | 0.4685574 | 0.3313201 | 5.04% | 0.5898000 | 8.05% | 0.7002118 | 0.4951245 | 6.35% | 0.3356667 | 4.23% | 0.3529764 | 0.24959200 | 3.13% |  |
| Cusp 2-3 | 0.3877333 | 5.93% | 0.4759711 | 0.3365624 | 5.07% | 0.4265333 | 5.67% | 0.5687228 | 0.4021477 | 4.79% | 0.4248667 | 5.11% | 0.5209740 | 0.36838430 | 4.39% |  |
| Cusp 2-4 | 0.4115333 | 7.48% | 0.5442299 | 0.3848287 | 6.64% | 0.3721333 | 5.80% | 0.4265108 | 0.3015887 | 4.53% | 0.3649333 | 5.74% | 0.4144325 | 0.29304800 | 4.40% |  |
| Cusp 2-5 | 0.3413333 | 3.92% | 0.4227869 | 0.2989555 | 3.35% | 0.4590769 | 5.00% | 0.5639615 | 0.3987810 | 4.06% | 0.3998333 | 4.16% | 0.5031764 | 0.35579950 | 3.55% |  |
| Cusp 2-6 | 0.3185000 | 3.86% | 0.3818832 | 0.2700322 | 3.25% | 0.5013333 | 6.15% | 0.5990690 | 0.4236058 | 4.66% | 0.2834000 | 2.91% | 0.2931822 | 0.20731110 | 2.14% |  |
| Cusp 2-7 | 0.1305000 | 3.98% | 0.1305086 | 0.09228353 | 2.80% | 0.2572000 | 7.54% | 0.3247331 | 0.2296210 | 6.08% | 0.2986667 | 8.06% | 0.3016930 | 0.21332920 | 5.68% |  |
| Cusp 3-4 | 0.3932000 | 6.09% | 0.4637065 | 0.3278900 | 4.95% | 0.3576667 | 4.95% | 0.4365338 | 0.3086760 | 4.20% | 0.4442000 | 6.21% | 0.5531816 | 0.39115850 | 5.31% |  |
| Cusp 3-5 | 0.3590000 | 9.07% | 0.4887228 | 0.3455792 | 8.40% | 0.2744615 | 8.21% | 0.3551234 | 0.2511102 | 6.44% | 0.2515833 | 6.54% | 0.3105263 | 0.21957530 | 5.71% |  |
| Cusp 3-6 | 0.3232500 | 5.05% | 0.3642688 | 0.2575769 | 3.94% | 0.3406667 | 6.22% | 0.3811137 | 0.2694881 | 4.56% | 0.2982000 | 5.13% | 0.3402796 | 0.24061400 | 3.99% |  |
| Cusp 3-7 | 0.2415000 | 3.86% | 0.2809172 | 0.1986385 | 3.09% | 0.4918000 | 6.73% | 0.6245176 | 0.4416006 | 5.67% | 0.3076667 | 3.86% | 0.3628760 | 0.25659210 | 3.26% |  |
| Cusp 4-5 | 0.2422667 | 4.54% | 0.3481594 | 0.2461858 | 4.37% | 0.2517692 | 4.93% | 0.3736176 | 0.2641875 | 4.52% | 0.3135833 | 5.60% | 0.4158200 | 0.29402910 | 4.70% |  |
| Cusp 4-6 | 0.1821250 | 5.24% | 0.2033350 | 0.1437796 | 4.31% | 0.2685000 | 8.77% | 0.3381319 | 0.2390953 | 6.46% | 0.1064000 | 2.41% | 0.1363378 | 0.09640539 | 2.01% |  |
| Cusp 4-7 | 0.3410000 | 13.74% | 0.3628457 | 0.2565707 | 9.96% | 0.2040000 | 7.15% | 0.2443907 | 0.1728103 | 5.47% | 0.1403333 | 3.70% | 0.1623874 | 0.11482520 | 3.04% |  |
| Cusp 5-6 | 0.1631250 | 4.24% | 0.1832345 | 0.1295663 | 3.41% | 0.1255000 | 4.06% | 0.1647346 | 0.1164850 | 3.74% | 0.1910000 | 6.74% | 0.2159903 | 0.15272820 | 4.89% |  |
| Cusp 5-7 | 0.2735000 | 3.90% | 0.2735041 | 0.1933966 | 2.70% | 0.3704000 | 4.64% | 0.4683396 | 0.3311661 | 4.10% | 0.3773333 | 4.53% | 0.4206186 | 0.29742230 | 3.57% |  |
| Cusp 6-7 | 0.1170000 | 2.37% | 0.1170000 | 0.08273149 | 1.66% | 0.2153333 | 3.44% | 0.2851888 | 0.2016590 | 3.23% | 0.0500000 | 0.69% | 0.0500000 | 0.03535534 | 0.48% |  |
