# Supplementary material for: Evaluating predictions of the patterning cascade model of crown morphogenesis in the human lower mixed and permanent dentition
Source: PLoS One. 2024 Jun 27;19(6):e0304455. doi: 10.1371/journal.pone.0304455 (PMC11210800; doi:10.1371/journal.pone.0304455)
Supplement: S1 Text — (DOCX) [file pone.0304455.s006.docx]

**Materials and Methods**

**Study Sample**

The study sample includes digital images and scans of modern human dental casts collected from Solomon Islander individuals over the course of the Harvard Solomon Islands Project (HSIP) [1]. The HSIP collected dental casts from individuals belonging to several language groups, most of which are currently housed at the Peabody Museum of Archaeology and Ethnology at Harvard University. In collaboration with the Peabody Museum, the Global Dental Phenomics Project has generated anonymized scans and images of participant dentitions, which were utilized as part of this study. The sample includes 124 individuals from the island of Malaita for whom dm_2_-M_1_ and/or RM_1_-LM_1_ pairs were present in the dental arcade, cusp tip placement was clearly discernible, and dental morphological trait expression could be determined. The study was deemed exempt from review by the University of Arkansas’ Office of Research Integrity and Compliance and the Institutional Review Board of Arizona State University pursuant to Federal Regulation 45CFR46(4)—Study 00007452. Loan and use of study materials were approved by the Harvard Peabody Museum and no personally identifying information was accessed as part of this research.

**Data Collection**

Dental data were collected from digital 3D scans of anonymized mandibular dental casts capturing the mixed or permanent dentition. All data were collected by the primary author (DEK), except deciduous morphology, which was collected by the senior author (KSP). Individuals were included in the study sample if the entire crown under observation had completed gingival eruption, secondary enamel knot location could readily be estimated based on cusp tip placement for all cusps, and morphological trait scores could be assessed based on cusp shape and size. Individuals with excessive cusp wear obliterating cusp tip location and accessory cusp morphology, as well as casts exhibiting casting errors (*i.e.*, bubbles, nodules) were excluded from the study. Whenever observable, the dm_2_-M_1_ metamere and the RM_1_-LM_1_ antimere pair were examined. Due to extensive dm_2_ wear and sample size concerns, examination of deciduous antimeres was not possible. For metameric comparisons in the mixed dentition, the dm_2_ least affected by dental wear was selected for analysis and the permanent M_1_ located on the same side as the dm_2_ was examined. Whenever the M_1_ on the same side as the dm_2_ could not be observed, values from its antimere were substituted. The data collection and analysis protocols employed in the current study closely follow those utilized by Hunter and colleagues [2], Skinner & Gunz [3], Moormann and colleagues [4], Paul and colleagues [5], and Ortiz and colleagues [6] to facilitate the contextualization of the results.

The 3D scans of mandibular casts were visualized in the 3D mesh processing software system MeshLab v2022.02 [7]. Cusp tips were marked for all cusps visible on the crown surface and the measuring tool was used to create a linear size reference in millimeters just above the crown. Including the size reference, a screenshot was taken of the crown from an occlusal view. This image was transferred to the Java-based image processing software ImageJ v.1.53, where intercusp distance and crown area data were collected [8]. Using the linear size reference included on the image, all measurements were calibrated to scale. Intercusp distances were collected between all cusps present on the crown using the line selection tool (S1 Fig). Tooth size dimensions were measured between margins at the maximum curvature points following established standards [9-11] (see S1 Fig). We utilized crown area (*i.e.*, occlusal surface area) as a proxy corresponding to developmental timeframe since previous studies found larger crowns to be characterized by extended periods of gestation [12-13]. Occlusal surface area was calculated by multiplying the 2D linear measurements of maximum buccolingual (BL) and mesiodistal (MD) diameter (OSA=BLxMD).

Dental morphological data were collected for LM_1_ and RM_1_ using the 3D digital scans visualized in MeshLab. For dm_2_, morphological data were collected through primary observation. A previous precision study using casted dentitions showed high correspondence in resulting datasets when comparing scores recorded from 3D scans to those collected via primary observation: mean error for lower accessory cusps ranged between 0.04-0.09 grades and percent concordance ranged between ~81-93% [14]. Data collection protocol for the permanent molars followed the Arizona State University Dental Anthropology System (ASUDAS) (Fig 3; S1 Table) [11, 15]. The ASUDAS quantifies dental morphological variation on an ordinal scale to approximate the underlying continuous genotypic distribution that culminates in the phenotype [16-17]. Morphological data were collected for cusp 5 (hypoconulid), cusp 6 (*tuberculum sextum*), and cusp 7 (*tuberculum intermedium*) (Fig 2). The “1A” grade of the cusp 7 scoring system was collected from the images; however, this manifestation corresponds to a shouldering of the metaconid, representing a *metastylid* rather than demarcating a true accessory cusp that emerges from the mesial ridge between the metaconid and entoconid (grades 1-4) [18]. The emergence of the “metastylid-type” cusp 7 precedes emergence of the “interconulid-type” grades, further supporting the idea that these are developmentally distinct structures [19]. Therefore, individuals exhibiting a 1A grade of cusp 7 were eliminated from statistical analyses associated with cusp 7. While the ASUDAS is designed to capture morphological trait variation in the permanent dentition, this system has been successfully applied to the deciduous dentition and was utilized in this study [14, 20-22].

**Statistical Methods**

All statistical analyses were conducted in RStudio v4.2.2 [23-24]. Inter- and intra-observer error was determined for a small subset of the original sample (15 individuals). DEK re-measured dimensions and re-scored morphological trait expression for permanent teeth approximately six months after initial data collection to establish intra-observer error rates. Inter-observer agreement was calculated for morphological data by comparing trait scores between DEK and CMS (cusp 6 and 7) and KSP (cusp 5) for the permanent dentition. Error rates for deciduous morphology have been consistently within an acceptable range [5, 14, 20-21, 25]. Cohen’s Weighted Kappa was utilized to identify agreement rates for dental morphological trait scores using the *irr* package [26]. This statistic highlights rater agreement by capturing the magnitude of score differences between raters/scoring sessions, weighing large disparities more heavily. Mean absolute error, mean absolute percentage error, and root-mean-square error rates were determined to evaluate intra-observer error for the continuous data using the *Metrics* package [27]. Technical error of measurement (TEM) and relative technical error of measurement (%TEM) were also calculated for the metric variables [28-30]. Beyond establishing measurement error, paired tests were applied to all metric data collected for the same variable at two different times to determine if discrepancies were significant (α<0.050).

Prior to statistical analysis, Shapiro-Wilk tests were run for all variables tested in metameric (dm_2_-M_1_) and antimeric (RM_1_-LM_1_) comparative analyses to evaluate if the continuous data were normally distributed. Paired t-tests were used whenever results of the Shapiro-Wilks tests were non-significant, while paired-sample Wilcoxon signed-rank tests were applied to all ordinal morphological data and metric data with a non-normal distribution. Relative intercusp distances (RICD) were calculated to eliminate crown size as a confounding variable by dividing the absolute intercusp distance (AICD) by the square root of the crown area (SQRTA) (*i.e.*, RICD = AICD/SQRTA).

Using the *MASS* package, proportional odds logistic regression was performed to determine if the expression of later-developing accessory cusps (*i.e.*, cusp 5, cusp 6, and cusp 7) (dependent variable) was influenced by the relative distance between earlier-developing cusps (RICD) or occlusal surface area (a proxy for large tooth germ size and extended crown growth period) as predicted by the patterning cascade model of crown morphogenesis [31]. Brant tests were conducted using the poTEST function from the *MASS* package to establish if the proportional odds regression models met the assumption of proportionality [31-32]. The assumption of proportionality in the context of the current study implies that the coefficients describing the relationship between levels of the response variable are equal and their regression lines are parallel. Using the *car* package, likelihood ratio chi-square tests were calculated to determine the significance of model fit (α≤0.05) [33]. Odds ratios were generated to ascertain the degree to which the independent variable of interest (RICD, tooth size) impacts the odds of forming an accessory cusp with more pronounced expression. This was achieved by dividing the model coefficient by 10 to scale the odds ratio to a 0.1-unit change and then exponentiating the quotient.

**References**

1. Harris EF. Anthropologic and Genetic Aspects of the Dental Morphology of Solomon Islanders, Melanesia. Ph.D. Dissertation, Arizona State University. 1977.
2. Hunter JP, Guatelli-Steinberg D, Weston TC, Durner R, Betsinger TK. Model of tooth morphogenesis predicts Carabelli cusp expression, size, and symmetry in humans. PloS One. 2010; 5(7): e11844.
3. Skinner MM, Gunz P. The presence of accessory cusps in chimpanzee lower molars is consistent with a patterning cascade model of development. Journal of Anatomy. 2010; 217(3): 245-253.
4. Moormann S, Guatelli‐Steinberg D, Hunter J. Metamerism, morphogenesis, and the expression of Carabelli and other dental traits in humans. American Journal of Physical Anthropology. 2013; 150(3): 400-408.
5. Paul KS, Astorino CM, Bailey SE. The Patterning Cascade Model and Carabelli's trait expression in metameres of the mixed human dentition: exploring a morphogenetic model. American Journal of Physical Anthropology. 2017; 162(1): 3-18.
6. Ortiz A, Bailey SE, Schwartz GT, Hublin JJ, Skinner MM. Evo-devo models of tooth development and the origin of hominoid molar diversity. Science Advances. 2018; 4(4): eaar2334.
7. Cignoni P, Callieri M, Corsini M, Dellepiane M, Ganovelli F, Ranzuglia G. Meshlab: An Open-source Mesh Processing Tool (Version 2022.02). In: Scrano V, De Chiara R, Erra U, editors. Eurographics Italian Chapter Conference; 2008. pp. 129-136.
8. Schneider CA, Rasband WS, Eliceiri KW. NIH Image to ImageJ: 25 years of image analysis. Nature Methods. 2012; 9(7): 671-675.
9. Sciulli PW. Evolution of dentition in prehistoric Ohio Valley Native Americans III. Metrics of deciduous dentition. American Journal of Physical Anthropology. 2001; 116(2): 140-153.
10. Hillson S, FitzGerald C, Flinn H. Alternative dental measurements: proposals and relationships with other measurements. American Journal of Physical Anthropology. 2005; 126(4): 413-426.
11. Pilloud MA, Kenessey DE, Vlemincq-Mendieta T, Scott GR, Philbin CS. Dentabase User Manual v1.0; 2022. University of Nevada, Reno.
12. Garn SM, Osborne RA, McCabe KD. The effect of prenatal factors on crown dimensions. American Journal of Physical Anthropology. 1979; 51(4): 665-677.
13. Garn SM, Osborne RH, Alvesalo L, Horowitz SL. Maternal and gestational influences on deciduous and permanent tooth size. Journal of Dental Research. 1980; 59(2): 142-143.
14. Paul KS, Stojanowski CM. Performance analysis of deciduous morphology for detecting biological siblings. American Journal of Physical Anthropology. 2015; 157(4): 615-629.
15. Scott GR, Irish JD. Human Tooth Crown and Root Morphology. Cambridge University Press; 2017.
16. Grüneberg H. Genetical studies on the skeleton of the mouse: IV. Quasi-continuous variations. Journal of Genetics. 1952; 51: 95-114.
17. Scott GR, Pilloud MA. Dental Morphology. In: Katzenberg, MA, Grauer, AL, editors. Biological Anthropology of the Human Skeleton. John Wiley & Sons, Inc; 2018. pp. 257-292.
18. Skinner MM, Wood BA, Boesch C, Olejniczak AJ, Rosas A, Smith TM, Hublin JJ. Dental trait expression at the enamel-dentine junction of lower molars in extant and fossil hominoids. Journal of Human Evolution. 2008; 54(2): 173-186.
19. Kraus, BS, Jordan, RE. The Human Dentition Before Birth. Lea & Febiger; 1965.
20. Paul KS, Stojanowski CM, Hughes TE, Brook AH, Townsend GC. Patterns of heritability across the human diphyodont dental complex: Crown morphology of Australian twins and families. American Journal of Physical Anthropology. 2020; 172(3): 447-461.
21. Paul KS, Stojanowski CM, Hughes T, Brook AH, Townsend GC. Genetic correlation, pleiotropy, and molar morphology in a longitudinal sample of Australian twins and families. Genes. 2022; 13(6): 996.
22. Paul KS, Stojanowski CM, Hughes T, Brook A, Townsend GC. The genetic architecture of anterior tooth morphology in a longitudinal sample of Australian twins and families. Archives of Oral Biology. 2021; 129: 105168.
23. R Core Team. R: A Language and Environment for Statistical Computing. R Foundation for Statistical Computing. Vienna, Austria; 2023.
24. Posit Team. RStudio: Integrated Development Environment for R. Posit Software. Boston, MA; 2023.
25. Paul KS, Stojanowski CM. Comparative performance of deciduous and permanent dental morphology in detecting biological relatives. American Journal of Physical Anthropology. 2017; 164(1): 97-116.
26. Gamer M, Lemon J, Singh IF. Package ‘irr’: Various Coefficients of Interrater Reliability and Agreement (Version 0.84.1). 2019.
27. Hamner B, Frasco M. Package ‘Metrics’: Evaluation Metrics for Machine Learning. (Version 0.1.4). R Foundation for Statistical Computing. 2018.
28. Kieser JA. Human adult odontometrics: the study of variation in adult tooth size. Cambridge University Press; 1990.
29. Kieser JA, Groeneveld HT. The reliability of human odontometric data. The Journal of the Dental Association of South Africa. 1991; 46(5): 267-270.
30. Ulijaszek SJ, Kerr DA. Anthropometric measurement error and the assessment of nutritional status. British Journal of Nutrition. 1999; 82(3): 165-177.
31. Venables WN, Ripley BD. Modern Applied Statistics with S. Springer: 2002.
32. Brant R. Assessing proportionality in the proportional odds model for ordinal logistic regression. Biometrics. 1990: 1171-1178.
33. Fox J, Weisberg S. An R Companion to Applied Regression. Sage Publications; 2018.
